# The effect of familiarity on behavioral oscillations in face perception

**DOI:** 10.1038/s41598-023-34812-6

**Published:** 2023-06-22

**Authors:** Xiaoyi Liu, David Melcher

**Affiliations:** New York University Abu Dhabi, Abu Dhabi, UAE

**Keywords:** Human behaviour, Attention, Perception

## Abstract

**Abstract:**

Studies on behavioral oscillations demonstrate that visual sensitivity fluctuates over time and visual processing varies periodically, mirroring neural oscillations at the same frequencies. Do these behavioral oscillations reflect fixed and relatively automatic sensory sampling, or top-down processes such as attention or predictive coding? To disentangle these theories, the current study used a dual-target rapid serial visual presentation paradigm, where participants indicated the gender of a face target embedded in streams of distractors presented at 30 Hz. On critical trials, two identical targets were presented with varied stimulus onset asynchrony from 200 to 833 ms. The target was either familiar or unfamiliar faces, divided into different blocks. We found a 4.6 Hz phase-coherent fluctuation in gender discrimination performance across both trial types, consistent with previous reports. In addition, however, we found an effect at the alpha frequency, with behavioral oscillations in the familiar blocks characterized by a faster high-alpha peak than for the unfamiliar face blocks. These results are consistent with the combination of both a relatively stable modulation in the theta band and faster modulation of the alpha oscillations. Therefore, the overall pattern of perceptual sampling in visual perception may depend, at least in part, on task demands.

**Protocol registration:**

The stage 1 protocol for this Registered Report was accepted in principle on 16/08/2022. The protocol, as accepted by the journal, can be found at:10.17605/OSF.IO/A98UF.

## Introduction

The brain functions through back-and-forth communications among different specialized regions, which is thought to be coordinated by neural oscillations at different frequencies. The theta (3–7 Hz) and alpha- (8–12 Hz) band oscillations, in particular, have been linked to visual perception^1–3^. Although the neural generators of these rhythms and the specific functions they serve are still topics of debate, researchers have in general agreed that they reflect large-scale inter-areal modulation effects (e.g., between more high-level cortex and the visual cortex) and connections within the visual processing pathways^4,5^.

In the last decade, human psychophysical studies have demonstrated rhythms in perceptual outcomes that mirror these neural oscillations by collecting perceptual judgements at densely distributed time points after a visual event^6–13^. While such fluctuations were initially reported in studies of detection of relatively simple stimuli, recent work has shown temporal effects also for complex stimuli. For example, theta-band behavioral oscillations have been reported in both rapid object categorization^8^ and face detection tasks^14^ that have utilized a dual-target rapid serial visual presentation (RSVP) paradigm. In this paradigm, two identical targets are embedded in a stream of distractors, with targets separated by a varying stimulus onset asynchrony (SOA). Because the previous and subsequent distractors in the stream will suppress the lingering neural signals evoked by the target, the RSVP paradigm has the unique advantage of allowing us to isolate the processing of a brief target. However, the underlying mechanisms of the observed behavioral oscillations in the dual-target RSVP paradigm still remain largely unknown.

The current project aimed to replicate our previous finding of a general theta-band rhythm in accuracy in face perception task as a function of the SOA (Hypothesis 1) (Table 1), which was obtained from an online study that allowed for a highly diverse sample in terms of age and nationality, across several different continents, but provided less control over experiment parameters such as testing environment^14^. Moreover, our design aimed to disentangle different theories of the reasons for behavioral oscillations by introducing a new within-subject independent variable—the familiarity of the face stimuli. While face processing is in general highly efficient for humans, research has consistently shown a much more robust representation for familiar than unfamiliar faces in our brain, reflected in superior identification especially under challenging conditions^15–17^, faster saccadic response^17^, larger neural responses^19^, increased processing speed and reduced decoding latency^20,21^, and beyond. In particular, the increased neural response and faster processing speed for familiar faces would predict interesting changes in the dynamics of the behavioral oscillation.

**Table 1:** Design table.

Question	Hypothesis (if applicable)	Sampling plan (e.g. power analysis)	Analysis plan
Is there a theta and/or alpha-band oscillatory temporal structure in face perception?	Hypothesis 1: There will be a significant fluctuation in accuracy at 3–12 Hz as a function of the SOA between the two targets in the dual-target trials		Fast Fourier Transform and sinusoidal fitting. Statistical significance indicated by a non-parametric bootstrap test with a cluster-based permutation test to control for multiple comparison. Rayleigh test of non-uniformity
Is the behavioral oscillation in face perception a result of top-down processes (e.g., attentional sampling, and/or predictive coding) or sensory sampling?	Hypothesis 2: If it involves top-down processes, the peak frequency of oscillation in the familiar trials will be higher than that in the unfamiliar trials	To be able to detect a significant peak in each condition, 55 participants are needed to reach a power level of 0.97	Fast Fourier Transform; Non-parametric bootstrap
(If there is an effect of familiarity) Is the behavioral oscillation a result of rhythmic attentional sampling or predictive coding?	Hypothesis 2.1: If it reflects rhythmic attentional sampling, then the effect of familiarity (faster oscillation in familiar than unfamiliar trials) will be observed Study 2 where familiar and unfamiliar trials are mixed together		Fast Fourier Transform; Non-parametric bootstrap
(If there is no effect of familiarity) Does familiarity have any effect on behavioral oscillations?	Hypothesis 2.2: If the processing of familiar face is more robust, the amplitude of behavioral oscillation at the peak frequency will be larger in familiar than unfamiliar trials		One-sided paired-sample t-test (alpha level: 0.05)
Does familiar faces evoke a stronger attention capture/more consistent gender prediction?	Hypothesis 2.3: If stronger, the phase-locking difference (PLD) will be significantly greater than 0		Inter-trial phase coherence (ITC) analysis. Permutation test
Are people better at perceiving familiar faces than unfamiliar faces?	Hypothesis 3: If better, the accuracy in familiar trials will be higher than that in the unfamiliar trials		One-sided paired-sample t-test (alpha level: 0.05)

**Figure 1:**
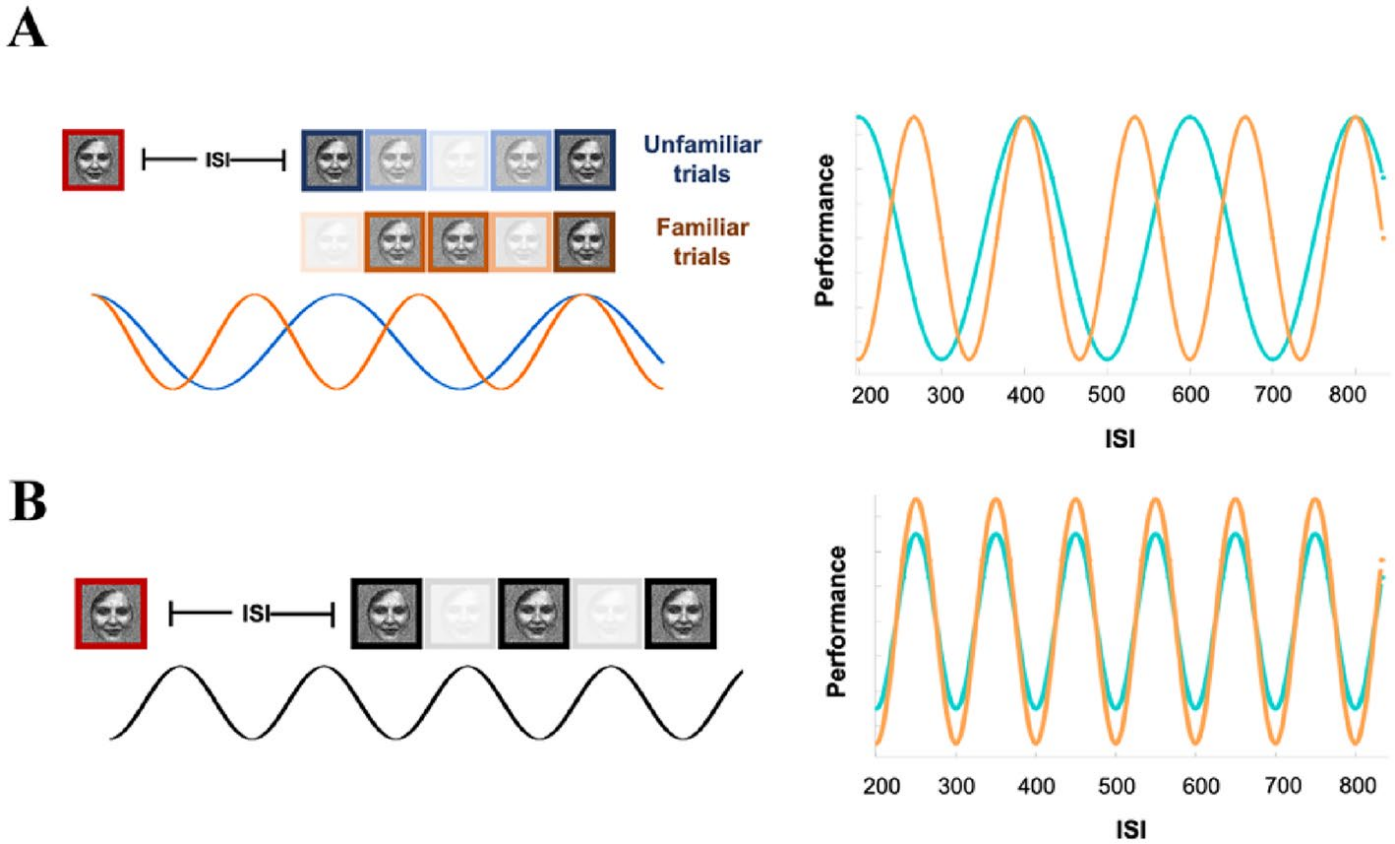
Hypothesized performance. (**A**) Behavioral oscillations as a result of top-down processes (Hypothesis 2). We expected that top-down neural modulation (left column) would oscillate faster in familiar (orange) than unfamiliar (blue) trials within theta-band frequencies. Right column shows the corresponding detrended behavioral performance as a function of SOA under the hypothesis. (**B**) Behavioral oscillations as a result of intrinsic sensory sampling at alpha frequencies (Hypothesis 2.2). In this case, face familiarity would not affect the sampling rate. However, because the processing of familiar faces is more robust, we expected to see a larger oscillatory amplitude in this condition. The transparency of the face images represents neural sensitivity to the stimuli. The face images used here were selected from the [64] (https://generated.photos/datasets).

The contrast between familiar and unfamiliar faces would enable us to critically test alternative hypotheses from different theories. Dividing the trials into familiar and unfamiliar blocks, we expected to see an effect of familiarity, in which the familiar block would not only yield better overall performance but also potentially show a faster oscillation than unfamiliar block (Hypothesis 2; Fig. 1A). This result would be based on the theory that behavioral oscillations are a result of top-down processes such as attentional sampling or predictive coding (for interpretation, see Table 2). Specifically, theories of attentional sampling propose that the functioning of early visual cortex (EVC) is modulated by the attention network at the frontoparietal regions through rhythmic neural activities at the theta-band^4,22^, giving rise to alternating moments of low and high perceptual responsiveness to visual inputs. In lab settings, researchers typically use a flash event to reset the attentional rhythm and observe the ‘attention spotlight’ to oscillate between multiple spatial locations at around 4 Hz^6,11,12^. Because this attentional sampling reflects a trade-off between exploration and exploitation, when the task is more difficult and requires more resources for internal processing (exploitation), researchers have observed a slower oscillation^9,23^. In our case, therefore, if the behavioral oscillation reflected attentional sampling, we would observe a slower oscillation at theta band in unfamiliar trials where the task was harder.

**Table 2:**
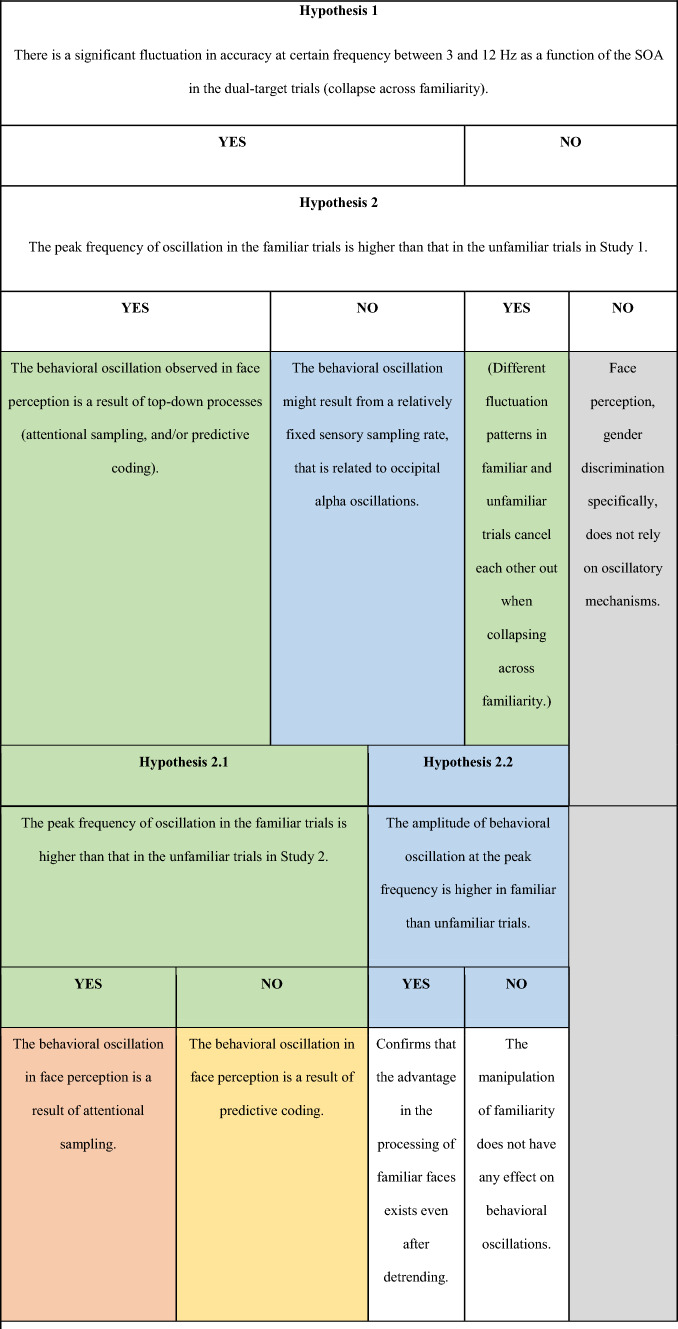

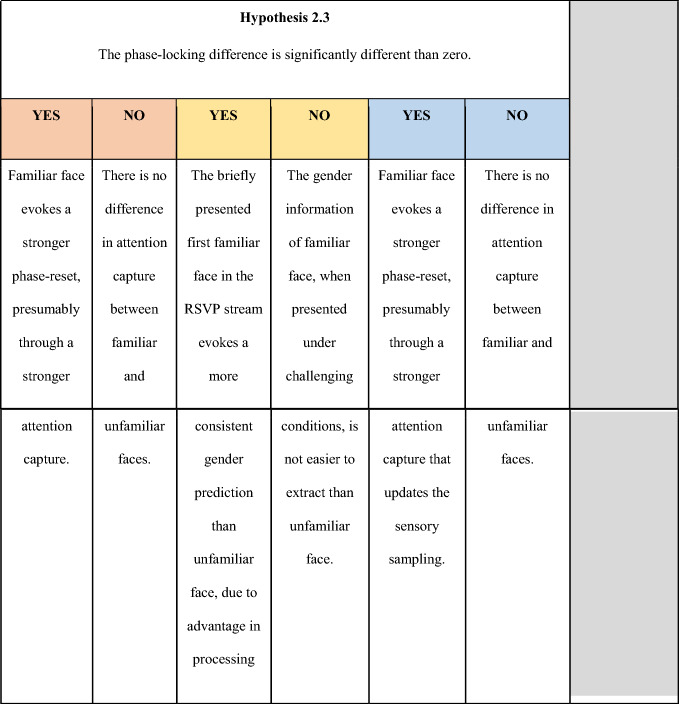
Interpretation Table.

In contrast, other theories suggest a role of predictive coding. Instead of viewing human vision as a passive sampling machine that automatically discretizes continuous input at a relatively constant rate, many variants of prediction theories claim that high-level cortical areas are constantly generating predictions about the incoming visual inputs and verifying these predictions via the backward connections to the EVC^24^. In line with this ‘active perceiver’ hypothesis, researchers have recently revealed a theta-band rhythmic interaction between the prefrontal cortex and mediodorsal thalamus in humans, the latter of which serves as a relay to update predictions stored in the cortex based on sensory inputs^25^. In priming studies, previous work has tested this idea by presenting a brief prime (e.g., an arrow, a face image) to activate multiple predictions about a following probe of the same kind^7,10,26^. These studies have revealed theta-band rhythmic fluctuations in reaction time as a function of the time lag between the prime and probe. In a recent neuroimaging study, Guo and colleagues^27^ used the same priming paradigm with a face/house discrimination task with fMRI. They found a similar 5 Hz oscillation in the decoding accuracy of voxels in the fusiform face area (FFA) and the parahippocampal place area, suggesting a possible origin of the behavioral oscillations at the category-selective regions. Because evidence has suggested that familiar faces elicit faster and more robust neural processing even when familiarity is irrelevant to the task^18,20^, this suggests that bottom-up neural signals will reach the prediction generation areas faster, and thus iterate faster. Therefore, if the behavioral oscillation reflected predictive coding, we would observe a similar effect of familiarity.

If an effect of familiarity was found, we then planned to conduct a second study to further differentiate the attentional sampling and prediction theories by mixing the familiar and unfamiliar trials together in the same block. We hypothesized a similar effect of familiarity as in Study 1 (Hypothesis 2.1), meaning that familiarity would trigger a fast automatic processing advantage. This fast adjustment is predicted by the attentional sampling theories. Alternatively, the effect of familiarity might only be observed in Study 1, in which the familiar and unfamiliar trials were presented in separate blocks. This pattern would be more consistent with a predictive coding hypothesis, which predicts that prior experience will slowly tune the oscillation function.

In contrast, the behavioral oscillation could result from a relatively fixed sensory sampling rate, which has been proposed to be related to occipital alpha oscillations^5^. This alpha-band sensory sampling has often been found embedded in slower theta-band attentional sampling. According to this idea, although attention monitors activities in visual system periodically through a theta-band rhythm, the visual system itself also samples from environment periodically at a faster alpha-band frequency^11,12^. For example, researchers have shown an intrinsic sensory sampling rate of 10–15 Hz by systematically modulating the signal-to-noise ratio of parts of the image at different frequencies^28,29^. If the sensory sampling rate is fixed, then the frequency of behavioral oscillation would be less susceptible to stimulus-related factors such as familiarity, and we would not observe a difference in oscillation frequency between familiar and unfamiliar blocks in Study 1 (Fig. 1B). However, given that the processing of familiar faces might still be more robust, this would lead to the specific hypothesis that the amplitude of behavioral oscillation at the peak frequency would be larger in familiar than unfamiliar blocks (Hypothesis 2.2). In other words, our manipulation would only influence the magnitude rather than the critical rate/frequency.

We also tested for other effects brought by the manipulation of familiarity (Table 1). Because familiar faces tend to evoke a stronger attention capture than objects and unfamiliar faces^18^, we predicted that familiar trials would be more phase-locked than the unfamiliar trials due to a stronger phase reset and phase alignment (Hypothesis 2.3). Finally, we expected to replicate previous finding of a prioritized detection of familiar faces in an RSVP stream^17^ by comparing the general gender discrimination performance (as measured with accuracy) between familiar and unfamiliar trials. We predicted a main effect of familiarity, with a higher accuracy in the familiar trials (Hypothesis 3).

## Methods

### Ethics information

Participants were recruited through the NYUAD SONA system. They must be aged between 18 and 40 years old, have normal or corrected-to-normal vision, and have no history of epilepsy or other neurological disorders to participate in the study. In addition, participants were asked how familiar they are with the identities in an online survey before the main experiment. They must be familiar with all 12 celebrity faces and unable to recognize the unfamiliar faces (see Appendix) to be included in the study. Participants received a small reimbursement for their time in terms of money (30 AED) or class credits (0.5 credit) for every 30 min they spend in the main experiment (around 90 min). The online survey was no longer than 20 min, and participants received 10 AED as compensation for completing the initial online survey (regardless of whether they were included in the in-person study). All participants were given enough time to read and sign the consent form and all aspects of the study will conform with international standards for ethical human subjects research. The experiment design was approved by the NYUAD Institutional Review Board (IRB).

### Design

**Figure 2:**
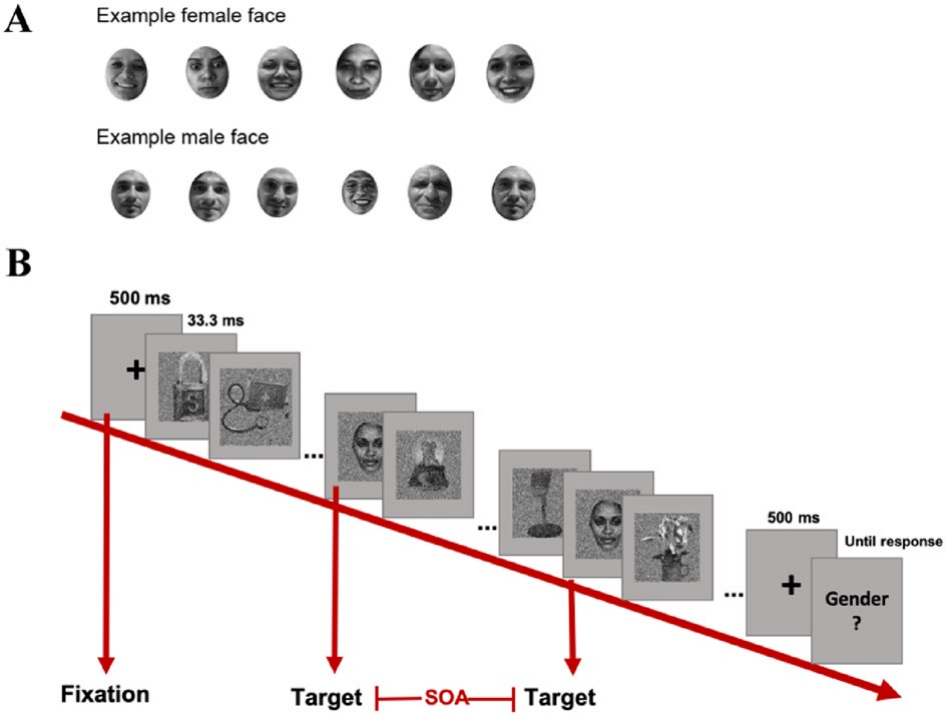
An illustration of the dual-target trials. (**A**) Sample face images. For each identity, we selected six instances of varied expressions and view angles. Note that images shown here are photos of lab research assistants, not from the actual stimulus set due to copyright. All rights reserved. Images were included here after obtaining written informed consent from the individuals depicted. (**B**) Thirty images were presented in an RSVP stream of 30 Hz (33.3 ms per image). Two identical face targets were embedded in the object distractors, separated by a SOA that varied between 200 ms to 833 ms in 20 steps of 33.3 ms. The first target always appeared randomly at either the 3rd, 4th, or 5th position in the stream. There were also one-target trials, where only the first target is presented. The face images used here were selected from the^64^ (https://generated.photos/datasets).

Images of non-face objects (baseline) and faces (targets) were presented in a dual-target RSVP paradigm. We used 4000 inanimate object images from the database used by Konkle and colleagues^30^. As a critical manipulation, we used 12 familiar faces (6 female) and 12 age (age when the photos were taken), gender, and ethnicity matched unfamiliar faces. The familiar faces were celebrity faces (e.g., famous actors and political figures; see Appendix) with images freely available on the internet. To ensure that participants were actually familiar with the supposedly familiar faces, participants completed an online survey before coming in for the main experiment. In the online pretest, they were presented with images of the 24 identities (different from the stimuli we used in the main experiment) and of 24 random unfamiliar identities and indicated their knowledge about him/her. Participants indicated how familiar with the faces by selecting from 1 (‘Yes, I know who this is.’) to 3 (‘I have never seen this face before.’). For the celebrity faces, participants also indicated their names and any knowledge they had regarding these people. The unfamiliar face images were people whose names might be famous but faces are unfamiliar to most people (e.g., writers, scientists, etc.). The purpose of including random unfamiliar identities that were not used in the study was to prevent participants from learning the unfamiliar identities. Participants were asked again about the identity of the familiar face images when they come in for the main experiment. To reduce the chance of learning the unfamiliar faces due to the repeated exposure, we selected six different images of the same identity as stimuli, such that each image was repeated for seven times (for example, see Fig. 2A; note that the images in Fig. 2A are not the actual stimuli for copyright issues. Images were included here after obtaining written informed consent from the individuals depicted.). All images were converted to gray-scale and resized to 256 pixels in width and height with Adobe Photoshop graphics software. Face images were further cropped with the ellipse tool with most hair and ear information cropped out. All images were histogram-matched for luminance through the SHINE toolbox^31^.

The study was coded with MATLAB PsychToolbox-3^32^. Stimuli were presented on a grey background (luminance: 128) on an ASUS monitor (1920*1080 screen resolution; 120 Hz refresh rate) at a viewing distance of approximately 90 cm. The EyeLink 1000 Plus eye-tracker (SR Research, Ontario, Canada) was used to ensure central fixation during the trials. We used a within-subject design. In each trial, participants were presented with a 500-ms fixation, followed by a stream of object images presented at 30 Hz. At the end of the image stream, participants were asked to indicate the gender of a face target (i.e., the gender discrimination task) by pressing keys (‘f’/‘j’ for ‘female’/‘male’ response; keyboard correspondence was counterbalanced across participants) on a keyboard. On the critical dual-target trials, two identical face targets were embedded and separated by a SOA that varied between 200 to 833 ms in 20 steps of 33.3 ms (Fig. 2B). This choice of SOAs was motivated by previous results with the dual-target RSVP paradigm^33^. The first target appeared randomly at either 3rd, 4th, or 5th position in the stream of 30 images. Each SOA condition was repeated for 48 times (2 familiarities × 12 identities × 2 repetitions). There were also 48 one-target trials, where only the first target was presented, to measure baseline performance.

In Study 1, Familiar and unfamiliar trials were divided into separate blocks. The order of the blocks was counterbalanced and predetermined depending on subject ID. For example, participants with an odd number ID ran the familiar block first and then unfamiliar block, while participants with an even number ID ran in the reversed order. In Study 2, everything would be kept the same except that familiar and unfamiliar trials would be randomly mixed together.

Before the main experiment, participants practiced with 72 one-target trials with varying levels of image contrast. A QUEST staircase procedure^34,35^ was implemented during the practice to estimate the image contrast at 60% accuracy for each participant. Target images were unfamiliar faces. All stimuli used in the practice trials were different identities from those in the main experiment. There were a total of 1008 trials divided into 16 blocks with self-paced breaks in between. Data collection and analysis were not performed blind to the conditions of the experiments.

### Sampling plan

To determine the sample size, we did a statistical power analysis by bootstrap resampling the data from 140 participants in our previous study^14^. Specifically, we (1) predefined a range of sample sizes from 20 to 60, (2) sampled participants from the original online dataset^14^ with replacement for 2000 times, (3) ran the same Fast Fourier Transform (FFT) analysis at each new dataset as in the current study, and (4) computed the power. The statistical power refers to the proportion of positive results (Bonferroni corrected *p* value < 0.05). In light of the results, our target sample size for each study was 55 participants, with a statistical power of 0.97 to detect an overall behavioral oscillation. A separate power simulation suggests that we would have a power of at least 80% to detect the difference in oscillatory frequencies in two conditions when the actual effect size is larger than 1.5 Hz.

We planned to first recruit 55 to complete the online survey. If according to the results of the online survey, less than 55 participants were eligible, we would continue to recruit until the number of eligible participants reached 55. Participants who were not able to complete the task due to technical issues or other personal reasons were excluded from the analysis. Participants who completed the task but whose accuracy in 200 ms SOA condition was below 50% were also excluded because the task should be the easiest in this condition. If a participant was excluded, then we would recruit and test an additional participant. This process continued until we had the full data of 55 participants. We did not analyze any other aspect of the data, other than checking that performance in the 200 ms SOA condition was above 50%, before we finished collecting the entire dataset. Participants who took part in Study 1 would not be eligible for Study 2.

### Data analysis

**Figure 3:**
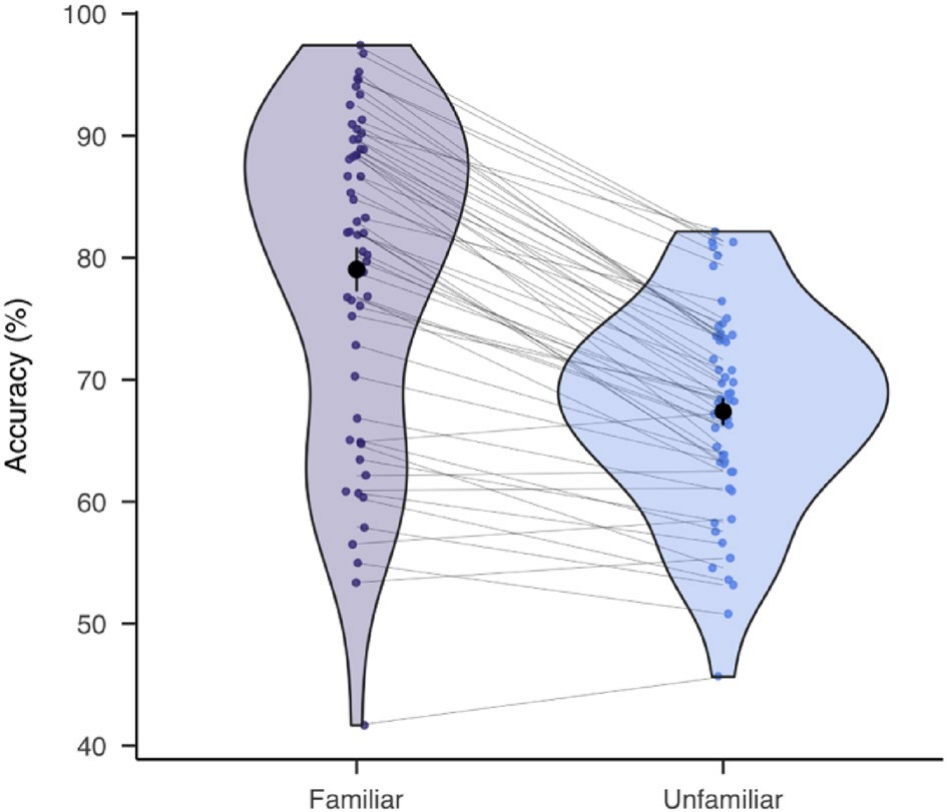
Better performance in familiar versus unfamiliar conditions. Accuracy on the group level (black dots) and individual levels (colored dots) in familiar (purple) and unfamiliar conditions (blue). Paired data were connected by the grey lines. The black lines around the mean dots represent one standard error of mean (SEM).

First, we computed the accuracy as the index for gender discrimination performance and calculated a one-sided paired-sample t-test (alpha level: 0.05) to compare the difference between familiar versus unfamiliar trials. For the main analysis, we focused on the accuracy in the dual-target trials. Average accuracy was computed for each SOA level for each participant. There was a slightly decreasing trend in the accuracy as the SOA increases (Fig. 3A), because targets presented within the same processing cycle tend to be integrated and enhance perception^36^. We fit an exponential decay function to the individual raw data and subtracted it from the raw data to get the detrended data:

$$f\left(x\right)={a}_{0}{e}^{-x/\tau }+c$$

where *x* is the SOA, ${a}_{0}$ the starting point, $\tau$ the time constant (that is, the variable describing the steepness of the decay) and $c$ the right-hand asymptote, or the baseline performance.

Then, a Fast Fourier Transform (FFT) was performed on the detrended data time course for each participant to convert the results from time domain to frequency domain. Zero padding was applied to achieve a frequency resolution of 0.1 Hz. We did not apply smoothing or any filters. The frequencies of interest were from 3 to 13 Hz, at which the theta- and alpha-band activities are typically defined. Next, we extracted the phase-locked sum (PLS) by summing the complex numbers of the FFT results of all participants, taking the absolute value, and dividing the result by participant number^9^:

$$PL{S}_{f}=\left(\frac{1}{n}\right){\sum }_{k}^{n}{A}_{\left(f,k\right)}{e}^{i2\pi {\theta }_{\left(f,k\right)}}$$

The $PL{S}_{f}$ is the phase-locked sum for frequency $f$, $n$ is the number of participants, $A$ and $\theta$ are, respectively, the amplitude and the phase angle of the complex vector, for each frequency $f$ and participant $k$. This computation allows us to test the amplitude of oscillations that are consistent in phase across participants. A permutation test was then performed to test the statistical significance of the result by randomly shuffling the SOA labels of the individual detrended data for 10,000 iterations and comparing the 95-percentile with the original amplitude for each frequency. We corrected for multiple comparisons by taking the maximum amplitude across all frequencies as threshold^10,37^. In light of the recent publication by Brookshire who proposes that the shuffling-in-time randomization procedure might conflate the results with aperiodic temporal structure in the time series^38^, we also verified any significant results with the new pipeline with a few modifications. First, we fit an autoregressive (AR(1)) model to the exponentially detrended data of each participant:

$${X}_{t}=c+ \phi {X}_{t-1}+ {\epsilon }_{t}$$

where ${X}_{t}$ is the accuracy at each time point, c is a constant, $\phi$ is the AR parameter, and $\epsilon$ is white noise. The fitted model was used to create 10,000 surrogate, pseudo-random dataset of each participant. On each surrogate dataset, we applied the same PLS computation, which created a null distribution of PLS based on random time series preserving the original aperiodic temporal component. We used the same multiple comparisons correction as before^37^. If there was a significant peak, we completed a Rayleigh test of non-uniformity for circular data to verify the phase concentration at the peak frequency (with MATLAB-based CircStat toolbox;^39^).

We also ran a sinusoidal fitting analysis on the detrended data to independently confirm any behavioral oscillations found in the FFT analysis. The fitting procedure was able to tell us how well our detrended data fit the sinusoidal wave at the frequency of interest. To this end, the adjusted R^2^ was computed. Statistical significance was again determined by a similar permutation test.

To test the effect of familiarity, we repeated the same analyses for the two familiarity conditions, analyzed separately. To evaluate any difference in the oscillatory frequencies, we resampled with replacement from the original data for 1000 times to get a distribution of the peak frequencies in each condition. Then we computed the Bootstrap estimates of standard error from the distributions, which were used to calculate the z-score of the difference between the two conditions^40^. The z-score was compared to the critical value of 1.64 (one-sided). We expected to see different peak frequencies, with a faster oscillation in the familiar condition. In the case of the null result, we planned to run an equivalence test with the two one-sided tests (alpha = 0.05, equivalence margin = 0.5) to confirm the result. If there was no difference in the peak frequency between familiar and unfamiliar conditions, we would simply compare the amplitude of behavioral oscillation (the product of 30 and the amplitude from the FFT results to compensate for the loss of the complex part of the signal) at the peak frequency (i.e., the effect size) between them with a one-sided paired sample t-test (alpha level: 0.05).

To test the phase-lock difference (PLD) between familiar and unfamiliar trials, we computed inter-trial phase coherence (ITC) for each familiarity condition by calculating the phase angle at the peak frequency for each participant and determining the average vector length with the CircStat toolbox. PLD was defined as: ITC_(familiar)_—ITC_(unfamiliar)_. Statistical significance of the PLD was determined with a permutation test by shuffling the trial label (familiar vs unfamiliar) of all trials for 10,000 iterations and computing a distribution of PLD. As before, we compared the value of the real PLD with the 95-percentile of the permutation result to get the *p* value (see^41,42^ for similar methods).

If there was a difference in peak frequency (i.e., the effect of familiarity), we planned to conduct Study 2, in which familiar and unfamiliar trials were mixed together within the same block and carry out the same analyses to test (1) the difference in gender discrimination performance between two trial types, (2) the general behavioral oscillation, (3) the difference in the oscillatory frequency between two trial types, and (4) the PLD.

## Results

### Participant information

Participants were recruited through the New York University Abu Dhabi (NYUAD) SONA system. As stated in our preregistered sampling plan, we continued recruiting participants until there were 55 qualified participants. In summary, we recruited 117 participants to complete the online survey, among which 57 qualified participants were invited to the in-lab experiment (Study 1). Two participants were excluded from the analysis because their discrimination accuracy in the 200 ms SOA condition was below 50%. All of the 55 participants (age: 19.85 ± 1.86 years old; 18 males) that were included in the final analyses reported normal or corrected-to-normal vision, and reported to have no history of epilepsy or other neurological disorders.

### Better performance in the familiar than unfamiliar blocks

Prior to the main task, participants indicated in an online survey how familiar they were with the face images. All participants who were selected for the behavioral task were more familiar with the famous (1.06 ± 0.11) (M ± SD) than unfamiliar identities (2.81 ± 0.13). The familiarity effect was reflected in the main task. One-sided paired-sample t-test showed a significant higher accuracy for the familiar (76.62% ± 1.34%) (M ± SE) than unfamiliar faces (65.43% ± 0.91%), *t*(54) = 12.51, *p* < 0.001. The trend was consistently found at the individual level in all but six participants (Fig. 3).

To confirm the familiarity effect in both the one- and dual-target trials, we ran a repeated-measures analysis of variance (ANOVA) with familiarity (familiar, unfamiliar) and target number (one, two) as within-subjects variables. Consistent with the t-test, there was a main effect of familiarity, *F*(1, 54) = 82.62, *p* < 0.001, *η*_*p*_^2^ = 0.60. We also found a main effect of target number, with enhanced performance for the dual-target (73.45% ± 1.20%) than one-target trials (68.60% ± 0.91%), *F*(1, 54) = 38.49, *p* < 0.001, *η*_*p*_^2^ = 0.42. The interaction between familiarity and target number was not significant. Thus, we could confirm that the familiarity effect was found in both single and double target trials.

### A theta-band fluctuation in gender discrimination accuracy

**Figure 4:**
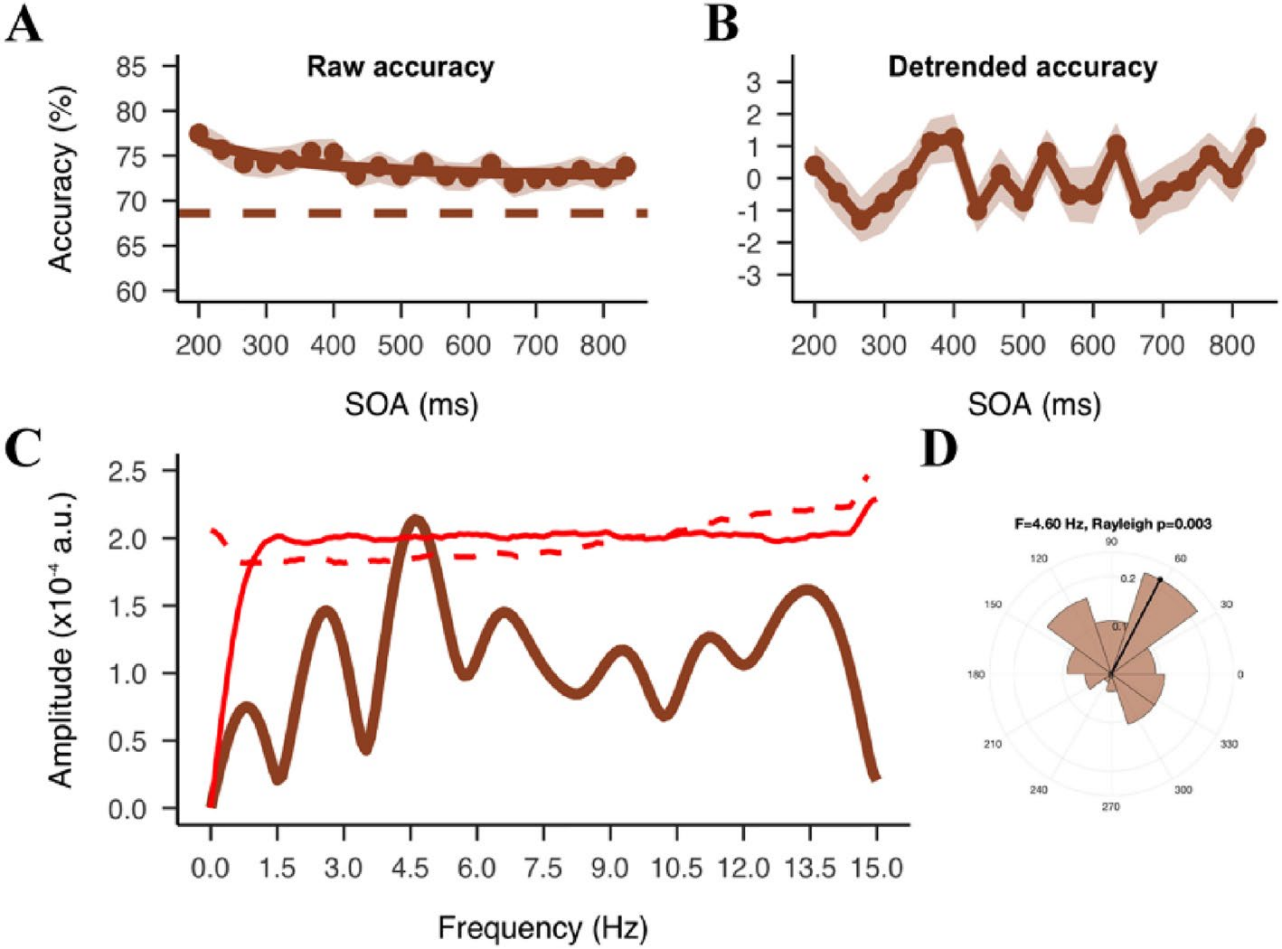
Results of all trials combined. (**A**) Raw accuracy in the dual-target trials as a function of the SOA between targets. Dots represent group average accuracy at each SOA. Shaded areas represent SEM. Solid lines show the exponential functions fit to the raw data. Colored dashed lines show the average accuracy in the one-target trials. (**B**) The average of individual detrended time courses. (**C**) Phase-locked sum of group spectra. The red solid line represents 95% percentile of permutations used to define significance of the peaks before multiple correction. The red dashed line represents similar permutation results but with the surrogate data suggested by Brookshire. (**D**) Polar histogram of the phase angle distribution for the highest peak at 4.6 Hz.

To evaluate the fluctuations in gender discrimination accuracy, an FFT analysis was performed on the detrended time course (Fig. 4B). We first computed the PLS at the pre-defined frequencies of interest from 3 to 13 Hz, with both familiar and unfamiliar trials combined. There was a significant peak at 4.6 Hz (Fig. 4C), *p*_*(shuffle-corrected)*_ = 0.038. The Rayleigh test revealed a strong phase coherence across participants at this frequency (*z* = 5.67, *p* = 0.003) (Fig. 4D). The first target onset had a robust phase reset effect that lasted at least 200 ms, such that performance was consistent across participants at the beginning of the behavioral oscillation at 4.6 Hz. The peak was also verified with the surrogate data suggested by Brookshire^38^ with a marginal significance, *p*_*(autocorrelation-corrected)*_ = 0.05 (Fig. 4C). To check the possibility of any significant peaks outside our frequencies of interest, we also computed the whole spectrum from 0 to 15 Hz, which was the limit of our effective frequency range (sampling rate of 30 Hz). This verified the 4.6 Hz peak as the only significant peak before correction. However, due to a large increase in the 95-percentile permuted PLS at the 15 Hz, the peak was not significant after corrected for the larger number of multiple comparisons (*p*_*(shuffle-corrected)*_ = 0.067, *p*_*(autocorrelation-corrected)*_ = 0.093).

**Figure 5:**
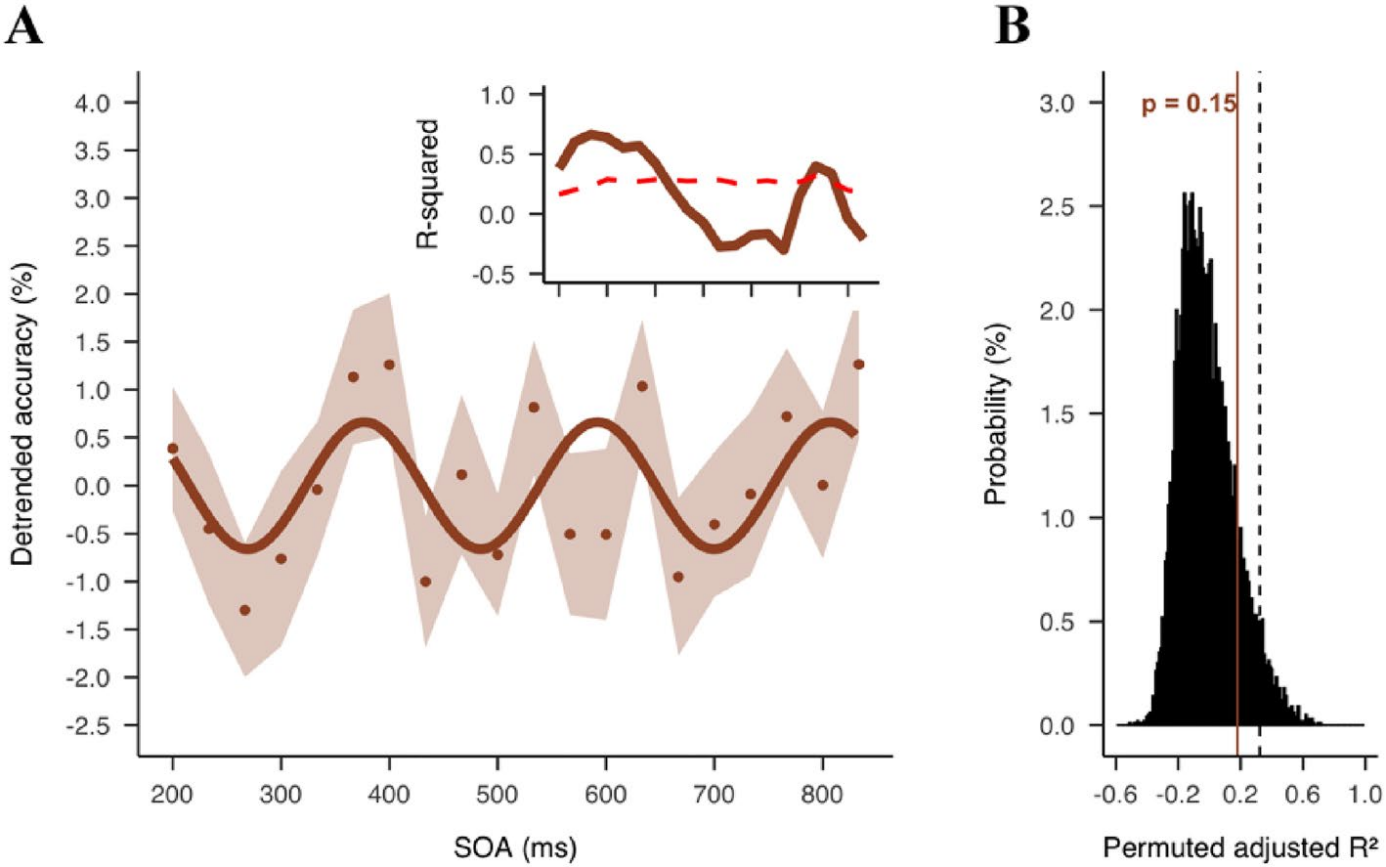
Sinusoidal fitting results. (**A**) Dots represent detrended accuracy at each SOA. Shaded area represents SEM. Solid line represents the sinusoidal function fitted to the data at 4.64 Hz. The small inset shows the fits (R-squared) at different time points. (**B**) Histogram of adj R-squared computed from each permutation (n = 10,000). Dashed vertical line represents 95% percentile of permutation distribution as threshold for significance. Brown solid line represents the observed adj R-squared.

Next, we ran a complementary sinusoidal fitting analysis on the group detrended data to confirm the behavioral oscillation at the group level. We found a best-fitting frequency at 4.64 Hz (adj *R*^2^ = 0.18, *p* = 0.15) (Fig. 5A), consistent with the PLS result. The non-significant result could be attributed to a decrease in sinusoidal fitting as the temporal distance from the reset event increased, a phenomenon that has been recorded in previous studies^43,44^. Therefore, as an exploratory analysis to quantify the decrease, we used a sliding time window method to compute the fit between segments of the original detrended time series and the best-fit sinusoidal function obtained from the sinusoidal fitting analysis. The time window had a length of 233 ms and was centered at the dual-target SOAs, so that we can compute a goodness of fit (*R*^2^) at each SOA. To compensate for the length difference when the time window was centered on the first and last few SOAs, we added zero paddings to the beginning and the end of the original time series. The same analysis was performed on the surrogate data to find the 95-percentile threshold, and the maximum *R*^2^ across the time series was used as the threshold. As shown in Fig. 5A, we found a significant fit at the first 200 ms of the time series (*p*_*(corrected)*_ < 0.05). The fit decreased to below the threshold 433 ms after the first target onset, and remained non-significant for the rest of the time series (except two significant time points at 733 and 767 ms).

We used the same method as Liu et al.^14^ to determine the effect size at the peak frequency. Instead of taking the PLS, we computed the amplitude of the FFT spectrum at 4.6 Hz on the individual data to obtain a distribution of the amplitude (Fig. S1). To test the relationship between FFT amplitude and the actual amount of variance, we simulated a time course fluctuating at 4.6 Hz with a true variance of 50% (-25% to 25%). We applied the same FFT analysis to the simulated data and found a peak at 4.6 Hz with an amplitude of 0.0167 arbitrary unite (a.u.). The 30 times discrepancy was due to zero-padding which would lead to a loss of signal (see Fig. S1). We therefore computed the amount of variance as the product of the FFT amplitude and 30. The result suggests that the fluctuation at 4.6 Hz can explain an average of 4.4% variance in the behavioral performance. The variance explained was slightly smaller than the previous study (6.6%)^14^, presumably due to a higher task demand in the current study (i.e., a gender discrimination task versus a face detection task in the previous study) that increased noise in the behavior response.

### Different oscillatory patterns for familiar and unfamiliar faces

**Figure 6:**
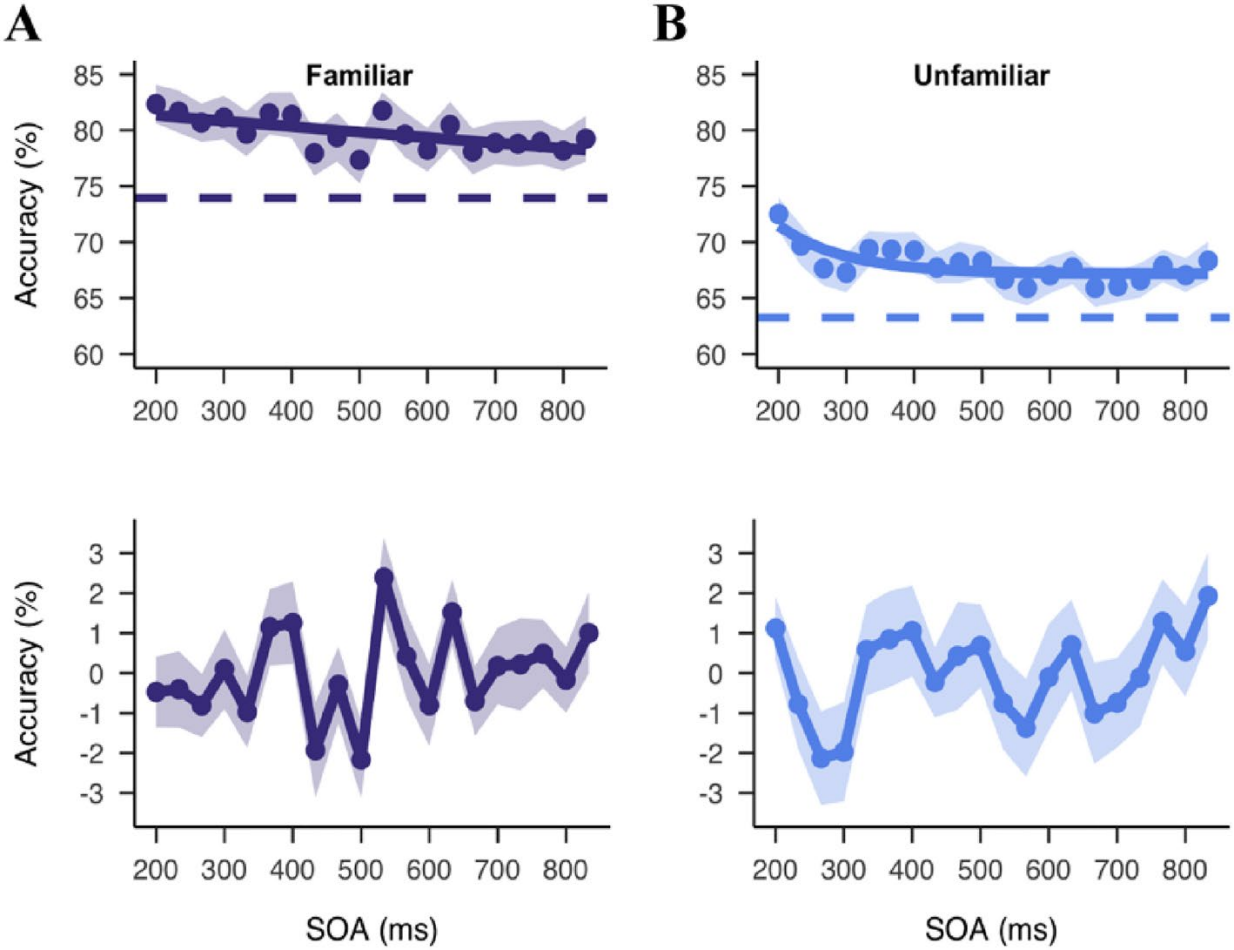
Time domain results for the familiar and unfamiliar conditions. (**A**) Raw (top) and detrended (bottom) accuracy as a function of the SOA in the familiar condition. Dots represent group average accuracy at each SOA. Shaded areas represent SEM. Solid lines show the exponential functions fit to the raw data. Colored dashed lines show the average accuracy in the one-target trials. (**B**) Raw and detrended accuracy in the unfamiliar condition.

**Figure 7:**
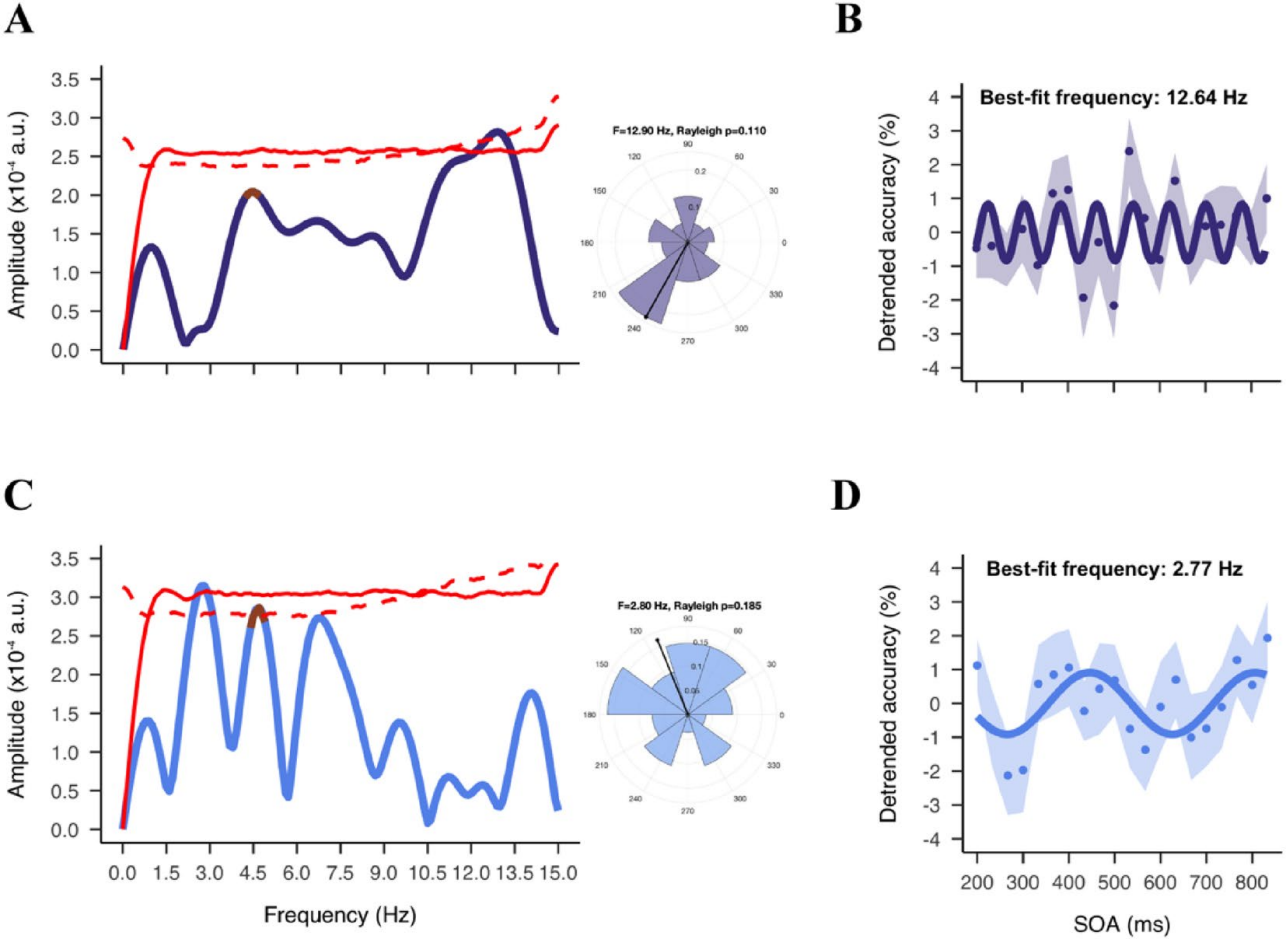
Fluctuations in the familiar and unfamiliar conditions. (**A**, **C**) Phase-locked sum of the familiar (**A**) and unfamiliar (**C**) spectrum and respective polar histogram of phase angle distributions at the highest peaks. The red solid line represents 95% percentile of permutations used to define significance of the peaks before multiple correction. The red dashed line represents similar permutation results but with the surrogate data suggested by Brookshire. (**B**, **D**) Sinusoidal fitting results in the familiar (**B**) and unfamiliar (**D**) conditions. Dots represent detrended accuracy at each SOA. Shaded area represents SEM. Solid line represents the sinusoidal function fitted to the data at 12.64 Hz (familiar) and 2.77 Hz (unfamiliar).

We next compared the frequency of the behavioral oscillation for the two conditions (familiar versus unfamiliar faces). We performed the same FFT analysis on the detrended data (Fig. 6) as above, but separately for familiar and unfamiliar face trials. Consistent with the result of the aggregated data, the peak at 4.6 Hz was found for both familiarity conditions (Fig. 7A & 7C), although neither reached significance with the smaller number of trials. This initially suggested that familiarity did not influence the rate of behavioral oscillation for faces. However, there was a difference in the overall pattern of behavioral oscillations between the two stimulus types. Specifically, within the pre-defined frequencies of interest, we found a highest peak at 12.9 Hz (*p*_*(shuffle-corrected)*_ = 0.029, *p*_*(autocorrelation -corrected)*_ = 0.043) for familiar trials and at 3 Hz for unfamiliar trials (*p*_*(shuffle-corrected)*_ = 0.064, *p*_*(autocorrelation -corrected)*_ = 0.089). This suggested that familiarity might have created a shift in behavior. However, these peaks were near the maximum (13 Hz) and minimum (3 Hz) frequencies under consideration. The a priori frequencies of interest were 3–13 Hz, at which the theta- and alpha-band activities are typically defined This raised the possibility that the true peaks were actually slightly lower or higher in frequency and just appeared to be at 3 and 13 since we were not looking for slower or faster frequencies in our analysis.

In order to better understand this difference for familiar and unfamiliar trials, we computed the whole spectrum (range 0–15 Hz) to determine where the real peaks were located. In the whole spectrum analysis, we found a marginally significant peak at 12.9 Hz, *p*_*(shuffle-corrected)*_ = 0.058 for familiar blocks (Fig. 7A). However, the phase coherence at this peak frequency did not reach significance (*z* = 2.20, *p* = 0.11). Sinusoidal fitting analysis confirmed a similar best-fitting frequency at 12.64 Hz on the group data (adj *R*^2^ = 0.10, *p* = 0.23) (Fig. 7B). For unfamiliar trials, on the other hand, there was a peak at 2.8 Hz, *p*_*(shuffle-corrected)*_ = 0.074, which was again not phase coherent across participants (*z* = 1.69, *p* = 0.19). Note that with the unfamiliar FFT spectrum, we also found two slightly smaller peaks at 4.6 Hz (as in the main analysis) and 6.8 Hz, both of which also reached significant phase coherence (4.6 Hz: *z* = 3.12, *p* = 0.044; 6.8 Hz: *z* = 3.64, *p* = 0.026) (Fig. 7C). The best-fitting frequency as indicated by the sinusoidal fitting analysis was 2.77 Hz (adj *R*^2^ = 0.14, *p* = 0.18) (Fig. 7D). Next, we compared the variance in behavioral performance that was accounted for by the oscillation at the peak frequency. As stated in the pre-registration, we ran a one-sided paired sample *t* test. The effect size was similar for the familiar (5.70% ± 0.41%) than unfamiliar blocks (8.22% ± 0.55%), *t*(54) = -3.73, *p* = 0.99. In light of this result, we ran a second two-sided paired sample t-test with an alpha level of 0.05, and confirmed a larger effect size in the unfamiliar than familiar blocks, *p* < 0.001 (Fig. S2).

We also compared the frequency difference between the two conditions using a bootstrapping procedure. We resampled the data 1000 times, and obtained the Bootstrapped distributions of the PLS in familiar and unfamiliar trials separately (Fig. S3). The difference in the mean peak frequencies between the two conditions was significant, *z* = 6.40, *p* < 0.001. The mean peak frequency was higher in the familiar trials (10.0 Hz) than unfamiliar trials (6.0 Hz). Thus, this finding was consistent with our second hypothesis. The results, however, should be taken with caution, as the fluctuations found in familiar and unfamiliar trials were not significantly phase coherent across participants. This lack of phase coherence suggests that either the effect of familiarity was not consistent across participants or might have been influenced by an additional, unknown factor.

We also tested whether there was a difference in the phase reset induced by the faces, since a more familiar face might have been more salient and thus caused a stronger reset. We found a small PLD between familiar and unfamiliar trials (ITC_(familiar)_ − ITC_(unfamiliar)_ = 0.025). The permutation test result, however, shows that the PLD was not different from zero (*p* = 0.27), suggesting that familiar faces did not induce a stronger phase reset than the unfamiliar faces.

## Discussion

Behavioral oscillations in the theta and alpha bands have been reported across many tasks in which the brain samples near-threshold signals across multiple spatial locations^2,6,12,22,45,46^ or multiple features at one location^11,47^. In a recent online study, we further revealed a theta-band fluctuation in the sampling of a continuous flux of object and face images^14^, demonstrating the impact of rhythmic sampling in more naturalistic vision tasks. Considering the constraints of online experimenting, the current study aimed to replicate the previous online finding and explore the effect of perceptual expertise on behavioral oscillations.

Consistent with our first hypothesis, we found a phase-coherent fluctuation in the theta band (at 4.6 Hz) in gender discrimination accuracy as a function of the SOA between the two target faces. This fluctuation accounted for 4.4% of the variance in discrimination performance. There are a growing number of studies providing evidence for a sampling rate in the theta frequency (4–7 Hz) for face perception. Several studies have demonstrated that face processing changes when stimuli are presented faster than about 3–6 images per second. In an fMRI study, for example, face and house images were presented at rates from 2.3 to 37.5 Hz, while temporal frequency responses were measured for various stages along the ventral processing stream^48^. While early visual areas showed peak responses for fast presentation rates, the fusiform face area (FFA) and scene selective regions (such as the para-hippocampal place area) peaked at rates of around 4–5 Hz. Similar findings have been reported in other fMRI studies using faces^49,50^. Further evidence for a sampling rate comes from studies using EEG, which have varied the presentation rate of faces and other complex images and measured the evoked response. For instance, Yeatman and Norcia^51^ varied presentation rate of faces and text images, alternated with noise, from 1 to 12 Hz and found a maximal face-selective response at 4 Hz. Thus, our main finding was consistent with our first hypothesis and the previous literature.

Our second hypothesis was that the familiar block would yield better overall performance and a faster behavioral oscillation. Indeed, there was a main effect of familiarity, with higher detection accuracy for familiar faces. We also found evidence implying a much faster fluctuation at higher frequencies (alpha) for familiar than unfamiliar face perception. In terms of the rate of the behavioral oscillation, a similar peak at 4.6 Hz was found for both familiar and unfamiliar trials. However, the largest/strongest peaks differed significantly between familiar (12.9 Hz) and unfamiliar (2.7 Hz) blocks. This pattern of results is partially consistent with the second hypothesis. The potential implications of these findings, with respect to our hypotheses, is considered in more detail below.

Although the current findings fit well with previous studies showing sampling in face processing at around 4–7 Hz, our results are also slower than the 7.5 Hz fluctuation found in our previous online study^14^. In the discussion of that paper, we discussed various reasons why our estimate might have been slightly higher than the actual sampling rate. The current study, in contrast, fits more closely with the 4–7 Hz that might have been expected based on the previous studies with faces and complex objects. Because most parameters of the two studies were the same, it is reasonable to attribute the discrepancy in the results to their primary difference: the specific task demands. In a detection task, as in the online study, people simply attend to the presence of a target among perceptually distinct distractors (e.g., finding a face in non-face objects). In contrast, the discrimination task used in the current study requires participants to compare the fine details of two stimuli within a category (e.g., female versus male faces). This additional computational requirement could affect the oscillatory frequency by adding more time to each ‘perceptual cycle’. Consistent with this theory, Dobs and colleagues^20^ have found that face presence information emerged around 20 ms earlier in the brain than its gender information. However, another factor might be that in order to prevent ceiling and flooring in behavioral performance, we titrated the stimulus contrast such that the discrimination accuracy in single-target unfamiliar trials would be around 60%. The true oscillation in gender discrimination performance might be masked by a decreased ability to detect the face target under such low contrast. Future studies could use other methods (e.g., face morphing) to control the discrimination performance without decreasing the face detectability.

The theta-band peak found here provides some evidence that the behavioral oscillations found in the dual-target RSVP paradigm may reflect rhythmic top-down modulations. In the spatial attention domain, for example, the 3–5 Hz sampling rate has been associated with a theta-band reweighting of the pulvino-cortical interaction^4,52^. The optimal phase of the theta activity is characterized by an increase in beta (15–30 Hz) and gamma (> 35 Hz) synchronizations at the frontal eye field (FEF) and the lateral intraparietal area, which are associated with suppressed motor functions and enhanced sensory processing respectively^52^. At the opposite phase, it is thought that the increase in alpha activity within the parietal cortex and the increased synchronization between the parietal cortex to the pulvinar enabled windows of attention to shift by temporally suppressing sensory processing^52^. In the current study, however, the targets appeared at a single location (the fovea) embedded in time among object distractors. While previous studies have shown similar theta-band fluctuation when sampling from multiple features^11,47^ or objects at the same location^27^, brain regions and pathways responsible for feature- and object-based attention may not fully overlap with those for spatial attention^53,54^. Feature-based attention, for instance, has been found to first emerge at the ventral prearcuate in the frontal lobe, rather than the FEF^53^. Future studies are needed to investigate the underlying neural mechanisms for rhythmic feature-based attentional sampling.

Our results also have implications to the computational models of populational neural responses over time. Whereas traditional models tend to explain neural responses with a linear function^55^, many recent models have achieved significant fit improvement by add a nonlinear summation component (e.g., an exponential modulation)^56,57^. Our results suggest that the models might be further improved by adding a rhythmic modulation at low frequency band. This could also potentially account for a temporal processing capacity of four to six items per second at areas along the ventral visual pathway^48–50^. For instance, Stigliani et al.^50^the largest neural response amplitude at the face-selective areas when face stimuli changed at 4 Hz, compared with 1, 2, and 8 Hz.

While the peak at 4.6 Hz was observed for both trial conditions separately, the largest/strongest peaks differed significantly between familiar (12.9 Hz) and unfamiliar (2.7 Hz) blocks. These results support the idea that the rate of perceptual sampling reflects contributions of different brain rhythms, rather than a single unitary sampling rhythm across all stimuli and tasks^58^. In a recent preprint, Kawashima and colleagues^37^ provided some preliminary evidence for this idea with an attentional blindness paradigm. They tested the detection of a second target following varied SOAs from the first target. Detection performance showed either an alpha- or a theta-band fluctuation as a function of the SOA, depending on the presence or absence of distractors in between^37^. ﻿Magnetoencephalography (MEG) recordings revealed a pronounced parietal theta activity in the no-distractor condition, reflecting periodic feedback modulations, but instead a dominant occipital alpha activity in the distractor condition, presumably reflecting an extra effort to suppress task-irrelevant inputs^37^. A similar principle has been observed in Ronconi et al.^3^, which reported that the frequency of the pre-stimulus neural oscillation that predicted behavioral performance, either theta or alpha, was related to the specific temporal integration window duration of the task. Even with the same task (e.g., visual search), researchers have shown that the oscillatory frequency increased with task complexity, presumably as a result of the increased attention cycles needed to complete the task^42^.

The difference in the strongest/peak rhythm between conditions in the present study might reflect a balance between feedforward and feedback processing^59–61^. When comparing the neural activation for the recognition of masked and unmasked objects, for instance, Bar et al.^62^ found that the orbitofrontal cortex was activated earlier than temporal object-selective region and facilitated object recognition. This top-down modulation, however, was only found when the object was masked or for low spatial-frequency images^62^. Using Granger causality analysis, they found that familiar face processing was dominated by a fast feedforward connectivity, whereas unfamiliar and phase-scrambled faces required the feedback communication from peri-frontal to peri-occipital area. It has been suggested that the speed of feedback communication is influenced by perceptual difficulty, with slower feedback under more challenging viewing conditions^61^. Considering this idea for the current study, in the familiar blocks, gender discrimination could rely on feedforward processing. The alpha-band behavioral oscillation we found could reflect either a dominant sensory sampling rhythm that operates independently from other processes^63^, or a faster top-down modulation. In spatial attention, for instance, it has been reported that attentional sampling was phase locked to ongoing alpha oscillations in prefrontal cortex when the need to shift attention was minimized^58^. In the current study, in which the familiar faces were easier (76.62% versus 65.43% in discrimination performance), this difference might have influenced the relative power of alpha versus theta bands.

Phase coherence is important when interpreting any peak frequency band differences and we did not find evidence of phase coherence at these peak frequencies that differed between familiar and unfamiliar blocks. Therefore, our familiarity effect results in the alpha band should be taken with caution. Phase coherence is a measure of the efficiency of the phase reset event. Significant coherence suggests that the brain rhythms responsible for the current task are in synchrony after the reset event and are consistent across individuals^9^. Previous studies typically used a flash^8^ or a spatially salient cue^6,12,45^ as a reset event. It is possible that the event in the current study (the first face target onset) was not perceptually salient enough to evoke a strong phase reset. As the PLD analysis reveals, the familiar faces did not evoke a stronger attention capture. For the same reason, we did not conduct the second preregistered study in which trial types would be mixed randomly in the same block to further disentangle the rhythmic attentional sampling and predictive coding theories. Future studies should implement a stronger attention reset event to capture a more reliable peak when the trials are mixed together.

Overall, the present study replicated our previous online finding^14^ showing fluctuations in performance as a function of the temporal interval between the face targets (face detection in the previous study, gender discrimination in the current study). This adds further evidence for a theta-band oscillatory temporal organization underlying visual perception for complex stimuli such as faces. We further showed that the rate of the behavioral oscillation was also modulated by task demands, consistent with the top-down modulation theories of rhythmic perceptual sampling. In the unfamiliar block where faces were perceptually less salient, we found a slower fluctuation in discrimination accuracy, implying a longer time for the feedback signals to return in this condition.

## Supplementary Information

Supplementary Information.

## Data Availability

We shared our raw data and materials at https://github.com/xiaoyiliuXL/familiar-rsvp.

## Code availability

All code will be made available (https://osf.io/ku8q9/) on acceptance of our Stage 2 manuscript.
