# Supplementary material for: The effect of familiarity on behavioral oscillations in face perception
Source: Sci Rep. 2023 Jun 22;13:10145. doi: 10.1038/s41598-023-34812-6 (PMC10287701; doi:10.1038/s41598-023-34812-6)
Supplement: Supplementary file 1 — Supplementary Information. [file 41598_2023_34812_MOESM1_ESM.pdf]

Supplementary Materials

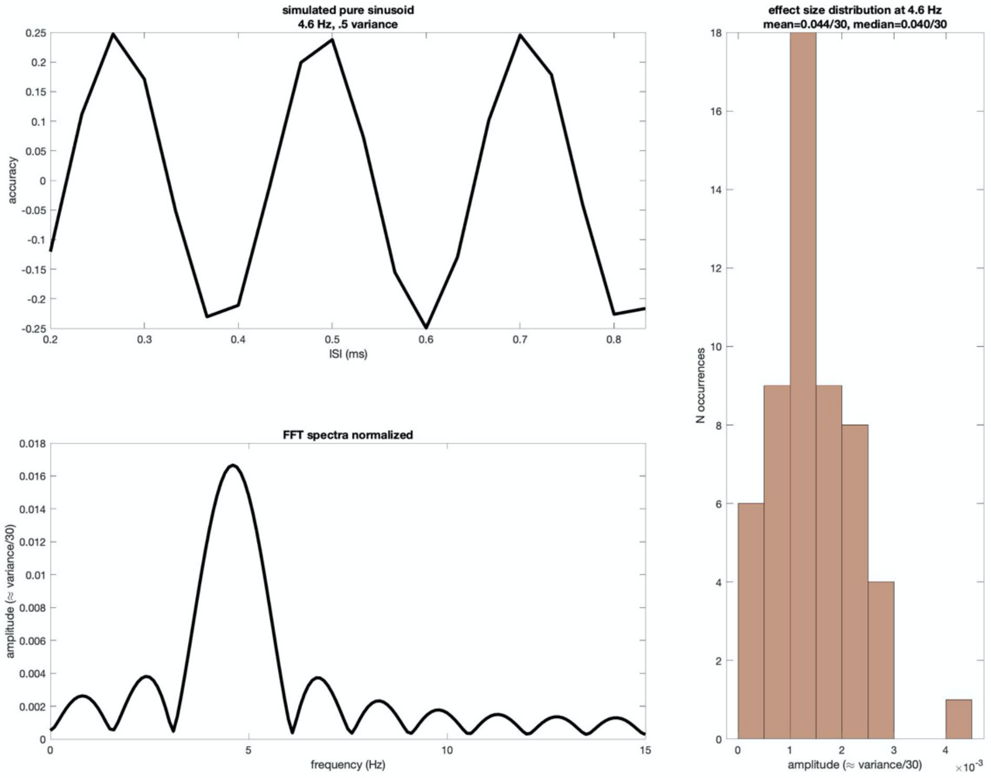

Figure S1: The variance in behavior performance explained by the 4.6 Hz fluctuation. The upper left inset shows a simulated, pure sinusoidal oscillation at 4.6 Hz sampled at our sampling frequency. The lower left inset shows the FFT spectra of the of the simulated sinusoid, whose amplitude have been multiplied by two to account for the removed complex part of the spectra. The true variance of the data (50%) was 30 times as large as the FFT amplitude due to the zero padding, which has led to a spread of power over several harmonic frequencies. The right inset shows the distribution of FFT amplitudes at 4.6 Hz computed as for the simulated sinusoid for the whole sample of participants. We then multiplied the FFT amplitude by 30 to obtain the mean variance (4.4%).

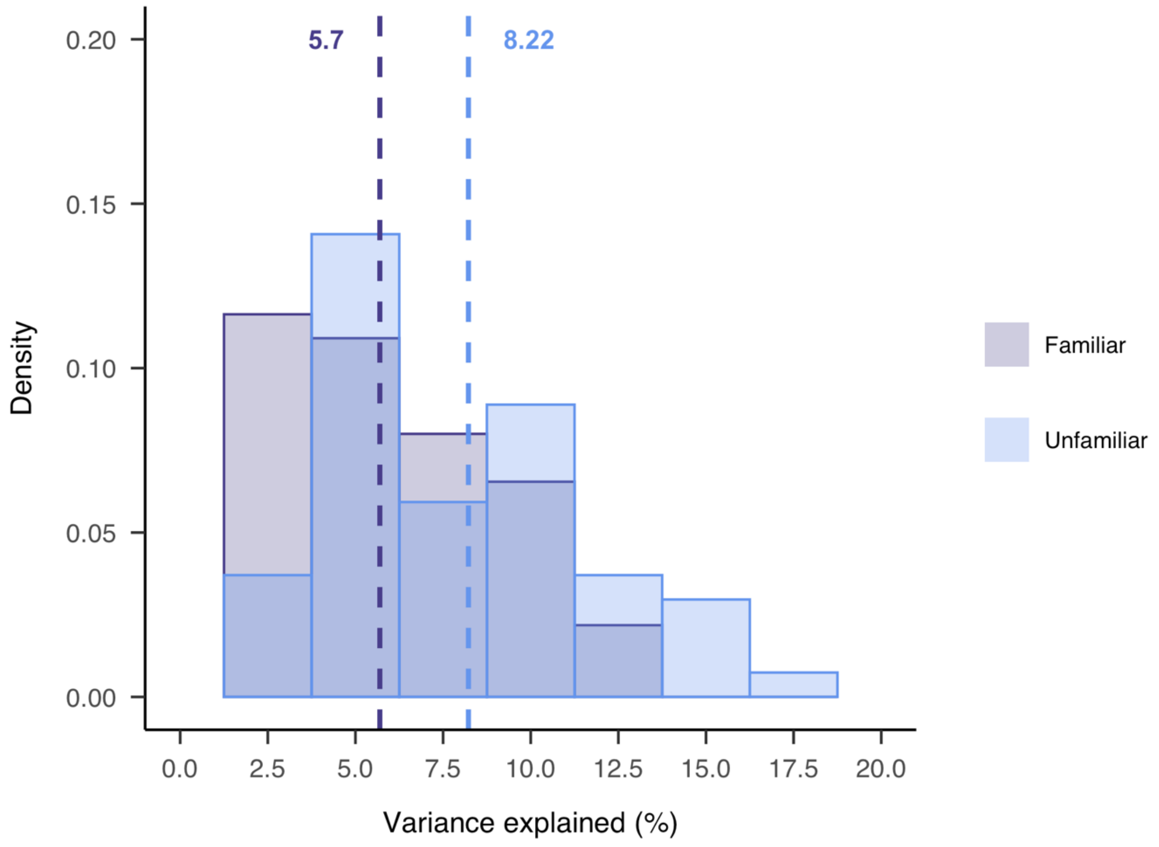

Figure S2: Distribution of variance in familiar (5.70% ± 0.41%) and unfamiliar (8.22% ± 0.55%) trials.

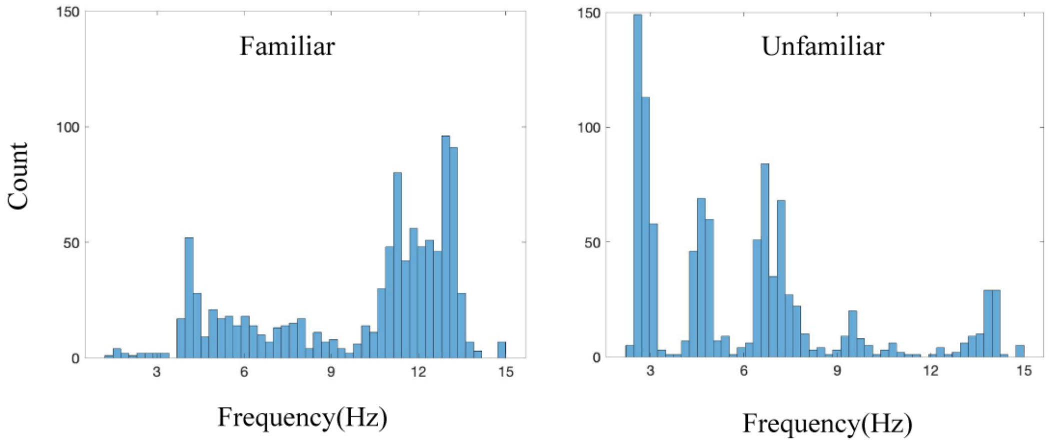

Figure S3: Bootstrap comparison of frequencies with highest phase-locked sum in familiar (left) and unfamiliar (right) conditions. As in our main findings, the Bootstrap result in the familiar condition shows a peak at around 12.9 Hz, with a mean at 10 Hz. In the unfamiliar condition, there are three peaks largely overlapping with the peaks we found in the main FFT analysis: 2.7 Hz, 4.6 Hz, 6.8 Hz, with a mean at 6 Hz.
